# Reproducibility of CRISPR-Cas9 methods for generation of conditional mouse alleles: a multi-center evaluation

**DOI:** 10.1186/s13059-019-1776-2

**Published:** 2019-08-26

**Authors:** Channabasavaiah B. Gurumurthy, Aidan R. O’Brien, Rolen M. Quadros, John Adams, Pilar Alcaide, Shinya Ayabe, Johnathan Ballard, Surinder K. Batra, Marie-Claude Beauchamp, Kathleen A. Becker, Guillaume Bernas, David Brough, Francisco Carrillo-Salinas, Wesley Chan, Hanying Chen, Ruby Dawson, Victoria DeMambro, Jinke D’Hont, Katharine M. Dibb, James D. Eudy, Lin Gan, Jing Gao, Amy Gonzales, Anyonya R. Guntur, Huiping Guo, Donald W. Harms, Anne Harrington, Kathryn E. Hentges, Neil Humphreys, Shiho Imai, Hideshi Ishii, Mizuho Iwama, Eric Jonasch, Michelle Karolak, Bernard Keavney, Nay-Chi Khin, Masamitsu Konno, Yuko Kotani, Yayoi Kunihiro, Imayavaramban Lakshmanan, Catherine Larochelle, Catherine B. Lawrence, Lin Li, Volkhard Lindner, Xian-De Liu, Gloria Lopez-Castejon, Andrew Loudon, Jenna Lowe, Loydie A. Jerome-Majewska, Taiji Matsusaka, Hiromi Miura, Yoshiki Miyasaka, Benjamin Morpurgo, Katherine Motyl, Yo-ichi Nabeshima, Koji Nakade, Toshiaki Nakashiba, Kenichi Nakashima, Yuichi Obata, Sanae Ogiwara, Mariette Ouellet, Leif Oxburgh, Sandra Piltz, Ilka Pinz, Moorthy P. Ponnusamy, David Ray, Ronald J. Redder, Clifford J. Rosen, Nikki Ross, Mark T. Ruhe, Larisa Ryzhova, Ane M. Salvador, Sabrina Shameen Alam, Radislav Sedlacek, Karan Sharma, Chad Smith, Katrien Staes, Lora Starrs, Fumihiro Sugiyama, Satoru Takahashi, Tomohiro Tanaka, Andrew W. Trafford, Yoshihiro Uno, Leen Vanhoutte, Frederique Vanrockeghem, Brandon J. Willis, Christian S. Wright, Yuko Yamauchi, Xin Yi, Kazuto Yoshimi, Xuesong Zhang, Yu Zhang, Masato Ohtsuka, Satyabrata Das, Daniel J. Garry, Tino Hochepied, Paul Thomas, Jan Parker-Thornburg, Antony D. Adamson, Atsushi Yoshiki, Jean-Francois Schmouth, Andrei Golovko, William R. Thompson, K. C. Kent Lloyd, Joshua A. Wood, Mitra Cowan, Tomoji Mashimo, Seiya Mizuno, Hao Zhu, Petr Kasparek, Lucy Liaw, Joseph M. Miano, Gaetan Burgio

**Affiliations:** 1 Mouse Genome Engineering Core Facility, Vice Chancellor for Research Office, University of Nebraska Medical Center, Omaha, NE USA; 2 Department of Pharmacology and Experimental Neuroscience, University of Nebraska Medical Center, Omaha, NE USA; 3 Transformational Bioinformatics, Health and Biosecurity Business Unit, CSIRO, North Ryde, Australia; 4 Texas A&M Institute for Genomic Medicine (TIGM), Texas A&M University, College Station, TX 77843 USA; 5 Department of Immunology, Tufts University School of Medicine, Boston, USA; 6 RIKEN BioResource Research Center, Tsukuba, Ibaraki 305-0074 Japan; 7 Department of Biochemistry and Molecular Biology, University of Nebraska Medical Center, Omaha, NE USA; 8 Departments of Anatomy and Cell Biology, Human Genetics and Pediatrics, Research Institute McGill University Health Center (RI-MUHC), Montreal, Canada; 9 Maine Medical Center Research Institute (MMCRI), Scarborough, ME USA; 10 Transgenesis and Animal Modeling Core Facility, Centre de Recherche du Centre Hospitalier Universitaire de Montreal (CRCHUM), Montreal, Canada; 11 Division of Neuroscience and Experimental Psychology, School of Biological Sciences, Faculty of Biology, Medicine and Health, Manchester Academic Health Science Centre, University of Manchester, AV Hill Building, Oxford Road, Manchester, M13 9PT UK; 12 School of Medicine, Indiana University, Indianapolis, IN 46202 USA; 13 South Australian Health & Medical Research Institute and Department of Medicine, University of Adelaide, Adelaide, Australia; 14 Transgenic Mouse Core Facility, VIB Center for Inflammation Research, Ghent, Belgium; 15 Department of Biomedical Molecular Biology, Ghent University, Ghent, Belgium; 16 Unit of Cardiac Physiology, School of Medical Sciences, Manchester Academic Health Science Center, University of Manchester, Manchester, UK; 17 High-Throughput DNA Sequencing and Genotyping Core Facility, Vice Chancellor for Research Office, University of Nebraska Medical Center, Omaha, USA; 18 University of Rochester Medical Center, Rochester, NY 14642 USA; 19 Division of Evolution and Genomic Sciences, School of Biological Sciences, Faculty of Biology, Medicine and Health, Manchester Academic Health Science Centre, University of Manchester, Manchester, UK; 20 Transgenic Unit Core Facility, Faculty of Biology, Medicine and Health, University of Manchester, Manchester, UK; 21 Department of Basic Medicine, Division of Basic Medical Science and Molecular Medicine, School of Medicine, Tokai University, 143, Shimokasuya, Isehara, Kanagawa 259-1193 Japan; 22 Department of Medical Data Science, Osaka University Graduate School of Medicine, Suita, Japan; 23 The University of Texas MD Anderson Cancer Center, Houston, TX USA; 24 Division of Cardiovascular Sciences, School of Medical Sciences, Faculty of Biology, Medicine and Health, The University of Manchester and Manchester Heart Centre, Manchester University NHS Foundation Trust, Manchester Academic Health Science Centre, Manchester, UK; 25 Department of Frontier Science for Cancer and Chemotherapy, Osaka University Graduate School of Medicine, Suita, Japan; 26 The Institute of Experimental Animal Sciences, Osaka University Graduate School of Medicine, Suita, Japan; 27 Centre de Recherche du Centre Hospitalier Universitaire de Montreal (CRCHUM), Montreal, Canada; 28 Children’s Research Institute Mouse Genome Engineering Core, University of Texas Southwestern Medical Center, Dallas, TX 75390 USA; 29 Manchester Collaborative Centre for Inflammation Research (MCCIR), School of Biological Sciences, Faculty of Biology, Medicine and Health, The University of Manchester, Manchester, UK; 30 Centre for Biological Timing, School of Medical Sciences, Faculty of Biology, Medicine and Health, University of Manchester, Manchester, UK; 31 Center for Matrix Biology and Medicine, Graduate School of Medicine, Tokai University, Isehara, Kanagawa 259-1193 Japan; 32 Department of Molecular Life Science, Division of Basic Medical Science and Molecular Medicine, School of Medicine, Tokai University, 143, Shimokasuya, Isehara, Kanagawa 259-1193 Japan; 33 Laboratory of Molecular Life Science, Foundation for Biomedical Research and Innovation, Kobe, Japan; 34 Department of Laboratory Animal Science, Support Center for Medical Research and Education, Tokai University, 143, Shimokasuya, Isehara, Kanagawa 259-1193 Japan; 35 Basic and Clinical Research, The Rogosin Institute, New York, USA; 36 Oxford Centre for Diabetes, Endocrinology and Metabolism, University of Oxford, Oxford, OX37LE UK; 37 Mouse Biology Program, University of California, Davis, USA; 38 Laboratory of Transgenic Models of Diseases and Czech Centre for Phenogenomics, Institute of Molecular Genetics of the Czech Academy of Sciences, Prague, Czech Republic; 39 College of Osteopathic Medicine, Marian University, Indianapolis, IN 46222 USA; 40 Laboratory Animal Resource Center, University of Tsukuba, Tsukuba, Japan; 41 Department of Gastroenterology and Metabolism, Nagoya City University Graduate School of Medical Sciences, Nagoya, Japan; 42 School of Health and Human Sciences, Department of Physical Therapy, Indiana University, Indianapolis, IN 46202 USA; 43 Lillehei Heart Institute Regenerative Medicine and Sciences Program, University of Minnesota, Minneapolis, MN USA; 44 Paul and Sheila Wellstone Muscular Dystrophy Center, University of Minnesota, Minneapolis, MN USA; 45 Department of Surgery, School of Medicine, University of California, Davis, Davis, USA; 46 McGill Integrated Core for Animal Modeling (MICAM), Montreal, Canada; 47 Department of Immunology and Infectious Disease, The John Curtin School of Medical Research, the Australian National University, Canberra, Australia

**Keywords:** CRISPR-Cas9, Mouse, Transgenesis, Homology-directed repair, Conditional knockout mouse, Floxed allele, Oligonucleotide, Long single-stranded DNA, Machine learning, Reproducibility

## Abstract

**Background:**

CRISPR-Cas9 gene-editing technology has facilitated the generation of knockout mice, providing an alternative to cumbersome and time-consuming traditional embryonic stem cell-based methods. An earlier study reported up to 16% efficiency in generating conditional knockout (cKO or floxed) alleles by microinjection of 2 single guide RNAs (sgRNA) and 2 single-stranded oligonucleotides as donors (referred herein as “two-donor floxing” method).

**Results:**

We re-evaluate the two-donor method from a consortium of 20 laboratories across the world. The dataset constitutes 56 genetic loci, 17,887 zygotes, and 1718 live-born mice, of which only 15 (0.87%) mice contain cKO alleles. We subject the dataset to statistical analyses and a machine learning algorithm, which reveals that none of the factors analyzed was predictive for the success of this method. We test some of the newer methods that use one-donor DNA on 18 loci for which the two-donor approach failed to produce cKO alleles. We find that the one-donor methods are 10- to 20-fold more efficient than the two-donor approach.

**Conclusion:**

We propose that the two-donor method lacks efficiency because it relies on two simultaneous recombination events in *cis*, an outcome that is dwarfed by pervasive accompanying undesired editing events. The methods that use one-donor DNA are fairly efficient as they rely on only one recombination event, and the probability of correct insertion of the donor cassette without unanticipated mutational events is much higher. Therefore, one-donor methods offer higher efficiencies for the routine generation of cKO animal models.

**Electronic supplementary material:**

The online version of this article (10.1186/s13059-019-1776-2) contains supplementary material, which is available to authorized users.

## Background

Gene inactivation through knockout alleles in a model organism such as mouse provides invaluable insights into the mechanisms of gene function and disease [1]. However, important challenges remain to successfully analyze the phenotypic impact of knockout (KO) genes in adult model organisms, as over 30% of the genes in mice are essential for development and cause embryonic lethality or neonatal subviability when deleted [2]. To overcome lethal phenotypes in gene-knockout models, conditional knockout (cKO) strategies have emerged [3]. cKO models usually involve the insertion of *LoxP* sites in introns flanking critical exon/s or (less commonly) in intergenic regions or flanking regulatory regions such as promoters and enhancers. When crossed with a *Cre* recombinase-expressing driver mouse, the Cre enzyme recognizes *LoxP* sequences and removes the intervening sequence. This leads to functional inactivation of the targeted gene in only the cells where the *Cre* is expressed and capable of targeting the DNA [3]. Generating a KO or cKO mouse previously required the use of embryonic stem (ES) cell-based homologous recombination with embryo manipulation, microinjection (MI), and assisted reproduction technologies (ART) [4]. These techniques were established in the 1980s and are still being used as gold standard methods. Based on this technology, large-scale efforts such as the Knockout Mouse Project (KOMP) [5] and the European Conditional Mouse Mutagenesis (EUCOMM) Program [6] have designed thousands of targeting constructs and generated modified ES cell clones for over 90% of coding genes. Using the ES cell clones, about 25% of mouse genes have been converted into cKO mice, all readily available and accessible in public repositories [7].

**Fig. 1:**
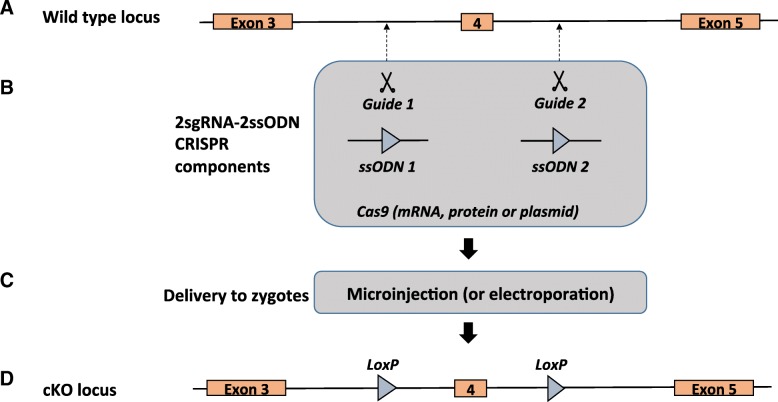
Schematic of two-donor floxing method of creating cKO alleles. **a** Wild-type locus showing exons 3, 4, and 5 of a hypothetical gene where exon 4 is chosen as a target exon for inserting *LoxP* sites. Guides 1 and 2 target introns 3 and 4, respectively. **b** CRISPR components for the two-donor floxing method Cas9 source. **c** Delivery method of CRISPR components into zygotes (microinjection or electroporation). **d** The cKO allele showing target exon (#4) with flanking *LoxP* sites

The recent emergence of genome editing technologies such as ZFN, TALENs, and CRISPR-Cas9, which can generate precise double-strand breaks in the genome, enables an improvement in the efficiency of gene targeting and has considerably facilitated the generation of genetically engineered animal models by direct injection of reagents into mouse zygotes [8]. Class 2 CRISPR system generates a precise double-strand break in the DNA via targeting with a simple-to-generate chimeric single guide RNA (sgRNA) and has become the most commonly used gene editing endonuclease system. The double-strand break leads to error-prone, non-homologous end joining (NHEJ) repair or homology-directed repair (HDR) under the guidance of a repair template [9, 10]. In an earlier study, a high success rate (16%) of targeting *LoxP* sites in *cis* was reported using two sgRNAs and two single-stranded oligonucleotides (ssODN) containing *LoxP* sites (herein referred to as “two-donor floxing method”) flanking a targeted critical exon (Fig. 1) [11].

We describe here a global community effort, from a consortium of 20 transgenic core facilities and programs across the world, to evaluate the efficiency of the two-donor method of generating cKO alleles and compare it with the recently described methods that use long single- or double-stranded DNA donors or "second *LoxP* insertion in the next-generation" approach.

## Results

### Generation of conditional alleles for *Mecp2* gene using the two-donor floxing method

We attempted to reproduce an experiment targeting the *Mecp2* gene, the locus for which creating floxed alleles using the two-donor floxing method was efficient at 16% [11]. We used the same sgRNAs and ssODNs described in the original report [11]. Three independent centers at the Australian National University (ANU) in Australia, University of Nebraska Medical Center (UNMC) in the USA, and the Czech Centre for Phenogenomics in the Czech Republic (IMG) performed these experiments on C57BL/6N inbred mice. The microinjected zygotes were cultured to blastocysts, and the genomic DNAs were analyzed by genotyping PCRs and Sanger sequencing (Table 1). Using a concentration mix of 10 ng/μl of Cas9 mRNA, 10 ng/μl of in vitro transcribed sgRNA, and 10 ng/μl of ssODN, we observed no successful targeting (i.e., correct insertion of two *LoxP* sites in *cis*-configuration) even though both sgRNAs cleaved target DNA as indicated by the presence of *indels* or integration of a *LoxP* site at the desired location, which varied from 13 to 33% (Table 1).

**Table 1:** Summary of the edited blastocysts for *Mecp2* gene from three different centers

	Zygotes injected	Blastocysts genotyped	Correctly targeted	Incorrectly targeted at the 5′ site (%)	Incorrectly targeted at the 3′ site (%)
Australian National University (ANU), Australia	106	51	0	11 *indels* and 6 *LoxP* correctly inserted (33%)	6 *indels* and 1 *LoxP* correctly inserted (13%)
University of Nebraska Medical Center (UNMC), USA	80	70	0	14 *indels* and 1 *LoxP* correctly inserted (21%)	21 *indels* (30%)
Czech Centre for Phenogenomics, Czech republic (BIOCEV/IMG)	40	28	0	8 *indels* and 1 *LoxP* correctly inserted (32%)	5 *indels* (18%)

Interestingly, we noted the occasional presence of mutations within the *LoxP* sites indicating illegitimate repair events at the target site or errors arising from the commercially synthesized donor DNAs. The frequency of successful targeting of two *LoxP* sites *in cis* was previously reported to be 16% [11], which we failed to replicate.

### A global survey of the generation of conditional alleles using the two-donor floxing method

To better assess the efficiency of the two-donor floxing method at other loci, we evaluated 56 additional loci in the mouse genome from a consortium of 20 institutions across Australia, Belgium, Japan, the USA, the UK, the Czech Republic, and Canada (Additional file 1: Table S1). This study was not pre-designed, rather, it constitutes data from the experiments performed at numerous laboratories that attempted using the two-donor floxing method to generate cKO mouse models. Of note, because the experimental conditions described in the original method were not producing desirable efficiencies, the laboratories in our consortium further modified the experimental conditions in an attempt to increase its efficiency. We report a compilation of such data from these laboratories, and thus, it naturally represents a “real-world situation” as it constitutes many diverse characteristics that were not pre-planned. Therefore, this dataset provided an opportunity to investigate the effect of many different parameters on the method’s efficiency.

The two-donor floxing data from the consortium was collected through a survey where investigators were requested to enter details of various parameters of the experiments in an excel spreadaheet file. For the easy presentation of the large dataset, we split the information from the single spreadsheet into 3 smaller spreadsheets. These data and the results are presented as Additional file 1: Table S1, Additional file 2: Table S2, and Additional file 3: Table S3, and the overall summary of the results is presented in Additional file 4: Table S4. Out of 17,887 zygotes (17,557 microinjected and 330 electroporated; see details below) zygotes, 12,764 (71.4%) were surgically transferred into recipient females. The recipient females gave birth to 1718 pups (9.6% of the microinjected/electroporated zygotes), of which only 15 pups (0.87%) contained the floxed alleles.

### Analysis of factors affecting the outcome of the two-donor floxing method

**Fig. 2:**
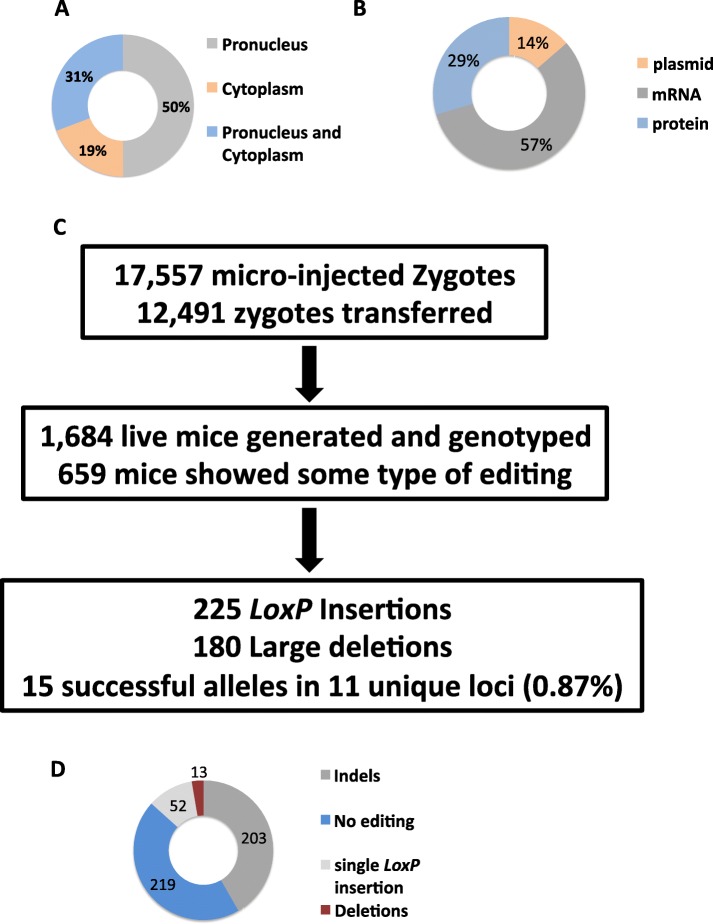
Quantitative assessment of the success of the two-donor floxing method. **a** Method of zygote injections (pronuclear, cytoplasmic, or both) for delivery of the CRISPR reagents used by reporting centers. Numbers indicate the percentage of the total zygotes microinjected or electroporated. **b** Form of the CRISPR reagents (mRNA, protein, or plasmid) delivered to the zygotes. Numbers indicate percentages. **c** Number of successfully edited alleles and correct *LoxP* insertions out of the total number of live-born pups from microinjected and transferred zygotes. Numbers indicate absolute numbers. **d** Types of editing observed among the live-born pups genotyped from a subsample from 25 loci. Numbers indicate absolute values

This large dataset enabled us to analyze the various factors affecting the outcome of the two-donor floxing method. These factors included different mouse strains, nature of loci (essential vs non-essential genes), distance between the two guides, different mode of deliveries (microinjection or electroporation), different reagent formats, different reagent concentrations, and differences in guide testing practices (some laboratories pre-test the guide RNAs, and some do not). A majority of projects were performed on a C57BL/6J background (39) whereas 18 projects used C57BL/6N background and 3 additional ones used a hybrid mouse background (B6C3HF1, B6SJLF1, FVBCD1F1). Statistical analysis of our data (Fisher exact test, *p* = 0.74) did not find any impact of strain background on the method’s efficiency. Of the 56 targeted loci (49 microinjected and 7 electroporated), 21 were ranked as essential genes based on early embryonic or postnatal lethality of homozygous knockout mice according to the mouse genome database http://www.informatics.jax.org [12]. Prior targeted deletions of 18 out of 56 of the loci were reported to generate homozygous mice that were viable into adulthood, and consequences of a null mutation at 17 loci were unknown. Together, this indicates the repartition between putative essential and non-essential targeted gene was in equal frequency (Fisher exact test, *p* = 0.76). Using a previously published machine learning method to predict gene essentiality in mice [13], we confirmed an equal frequency of essential and non-essential genes in our dataset (Fisher exact test, *p* = 0.99) The distance between sgRNA varied from 250 bp to 1.1 Mb with a median of 2 Kb. Single exons to entire genes or regulatory genomic regions (Additional file 1: Table S1) were floxed. We investigated whether the distance between sgRNA is critical for the likelihood of the success of the two-donor floxing method. We failed to find such evidence in our dataset (Kruskal-Wallis rank-sum test, chi-squared = 32, *p* = 0.42), although the sample size was too low to form a conclusion (Cohen’s effect size *d* = 0.40 with power 1-beta = 0.27). Of the 56 loci, 49 loci were microinjected (Additional file 2: Table S2) and 7 loci were electroporated (Additional file 3: Tables S3). Among the microinjected zygotes for 49 loci with 53 independent designs, significantly higher numbers of zygotes was microinjected to the pronucleus alone (26/53) rather than the cytoplasm alone (10/53) or both pronucleus and cytoplasm (17/53) (Fischer exact test *p* = 0.004), which is consistent with the current practice in most transgenic core facilities (Fig. 2a). Different forms of CRISPR reagents (synthetic or in vitro transcribed sgRNA, Cas9 mRNA or Cas9 protein, or sgRNA- and Cas9-expressing plasmid) were used (Additional file 1: Table S1). The majority of the projects used in vitro transcribed mRNA (35/59) at various concentrations varying from 10 to 100 ng/μl of Cas9 mRNA (Fig. 2b) and from 10 to 50 ng/μl sgRNA. ssODN were delivered at a concentration varying from 10 to 200 ng/μl. In 18 instances, Cas9 was delivered as a protein with a concentration varying from 10 to 75 ng/μl. Sixty-seven percent (41 pairs out of 61 pairs) of sgRNAs were tested in an in vitro cleavage assay prior to zygotic injection. We found no differences in editing efficiencies between the tested and the non-tested sgRNA sets [5′ guides: Kruskal-Wallis rank-sum test, chi-squared = 0.004, *p* = 0.94; 3′ guides: Kruskal-Wallis rank-sum test, chi-squared = 0.2, *p* = 0.65]. Interestingly, for 6 loci, Cas9 and sgRNAs were delivered in the form of a chimeric sgRNA-SpCas9 plasmid (pX330) at a concentration of 5 ng/μl. We sought to determine whether the forms of reagent delivery such as plasmid, ribonucleoprotein (RNP), or mRNA would have an effect on the overall efficiency in targeting using the two-donor floxing method. We failed to find such evidence (Fisher exact test *p* = 1).

Recently, electroporation of zygotes has been developed as an efficient method for generating knockout, point mutations, tagged, or conditional alleles [14–20]. From our consortium, three laboratories and programs surveyed the likelihood of success of the method. For seven loci surveyed, we noted success in inserting a single *LoxP* allele (Additional file 3: Table S3) from the analysis of blastocysts or live mice for two out of the seven loci. In contrast, we noted a relatively high frequency of large deletions and *indels* (up to 39% of large deletions) indicating a successful editing. However, none of the loci showed two *LoxP* sites inserted in *cis* in the offspring, suggesting that the delivery of CRISPR reagents by electroporation does not make a statistical difference in obtaining a desired outcome from the two-donor floxing approach, although the large numbers of embryos that can be manipulated allow for the recovery of a very small number of correctly targeted alleles.

**Fig. 3:**
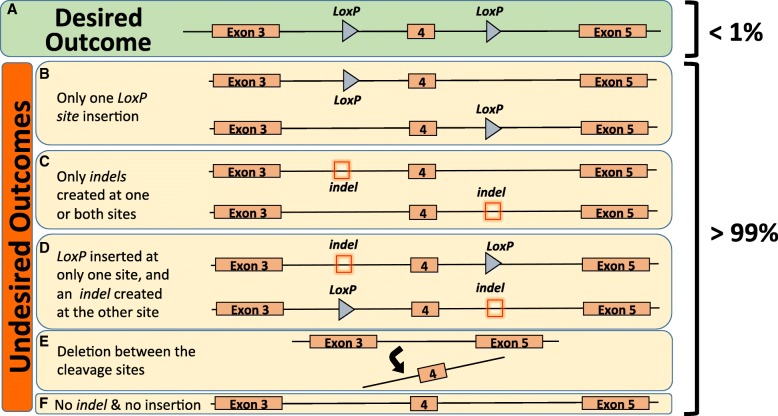
Desired and undesired outcomes of the two-donor floxing method. **a-f** Wild-type locus showing exons 3, 4, and 5 of a hypothetical gene where exon 4 is chosen as a target exon for inserting *LoxP* sites. **a** Desired outcome showing a floxed allele. Overall occurrence was < 1%. **b**–**f** Various undesired outcomes including only one *LoxP* site insertion (**b**), only *indels* created at one or both sites (**c**), combination of *LoxP* insertion and *indels* (**d**), deletion between the two cleavage sites (**e**), and no *indel* or no insertion events (**f**)

Next, we hypothesized that the success in generating floxed alleles using the two-donor floxing approach may depend on the factors such as (i) sgRNA efficiency, (ii) simultaneity in *LoxP* insertion, or (iii) the concentration of the Cas9, sgRNA, and ssODN reagents. To gain insight into these possibilities, we further analyzed data from the 56 loci (Additional file 2: Table S2, Additional file 3: Table S3). Note that the offspring for 54 loci were analyzed at the postnatal stage (Additional file 2: Table S2, Additional file 3: Table S3) whereas 2 loci were analyzed at the blastocyst stage (Additional file 3: Table S3). In some cases, the loci were further analyzed to assess guide cleaving activity. Of the 1684 founder mice, 676 (40%) carried editing events. Two hundred seventy-three mice (16%) showed some type of editing (*indels* and/or substitutions), and 225 (13%) and 180 (11%) mice harbored a single *LoxP* insertion or deletions between the two cleavage sites, respectively (Fig. 2c). The mice for 25/56 loci were further assessed for additional editing events including large deletions (Fig. 2c). Of the 487 founder mice analyzed (from those 25 loci), 219 (45%) 203 (41%), 52 (10.7%)%, and (13) 2.7% samples contained no editing, *indels*, single *LoxP* insertions, or large deletions, respectively (Fig. 2d). From the 1684 animals analyzed, only 15 mice (0.87%) were correctly targeted with intact *LoxP* sites in the *cis*-configuration (Additional file 2: Table S2). Out of the 56 loci, only 11 loci were successfully targeted (19.6%). The average number of zygotes needed to generate 1 correctly targeted animal was 1192. The essentiality of the genes had no impact on the likelihood of success of the two-donor floxing (4/23 success in targeting for embryonic or postnatal lethality vs 5/18 for viable homozygous mice and 2/15 for unknown embryonic or postnatal lethality, Fisher exact test *p* = 0.27). We also noted from our data, among the 56 loci analyzed, 14% showed deletions between the 2 target sites for Cas9 cleavage. We also noted a relatively high occurrence of single *LoxP* insertions for > 20% of the mice genotyped (from all loci) and a few instances of *trans*-*LoxP* insertions (on different alleles, reducing the probability for correct insertion of the *LoxP* sites) (Fig. 3). We therefore hypothesized that the success of this approach depends on the combined efficiency of the sgRNA and the likelihood of *LoxP* insertion at both sites to enable two *in cis* HDR events to occur simultaneously. To assess this postulate, we performed a generalized linear regression analysis to model the relationship between Cas9, sgRNA concentration, sgRNA cleavage efficiency, the distance between *LoxP* insertions, and frequency of *LoxP* insertions, with success of the two-donor floxing method as a positive outcome. The analyses are summarized in Table 2. The efficiency of *LoxP* insertions at both 5′ and 3′ sites appears to be the best predictor for the likelihood of success of the two-donor floxing method, accounting for over 80% of the total variance. However, this predictor was not significant in our linear regression model. Additional predictors such as sgRNA efficiency or efficiency in 5′ or 3′ insertion of *LoxP* explained approximately 15% of the total variance, but none of these predictors was significant in our model. The concentration of Cas9 mRNA accounted for less than 0.1% of the total variance but was statistically significant (*p* < 0.01) in the generalized linear regression model as a predictor for the success of the two-donor floxing method. However, the success of the two-donor floxing method was marginally correlated with an increase of Cas9 mRNA concentration (*r*^2^ Pearson = 0.27, *p* = 0.08). From our analysis, the sample size of the successful *LoxP* insertions in *cis* was too small to definitively rule out any other predictors (Cohen’s effect size *d* = 0.4, power 1-beta = 0.41). We reasoned that the analysis of only the loci where *LoxP* insertion events were observed may better predict the likelihood of the success of this approach (even though those insertions were not in *cis*-configurations). To test this, we performed a statistical analysis only on those loci containing *LoxP* sites and excluded the loci lacking *LoxP* insertions in either of the guide cleavage sites. We identified 28 such loci (from a total of 56 loci). We did not find any difference in predicting the outcome to the previous analysis with all loci (data not shown). Together, these results suggest that the presence of two simultaneous recombination events seemed to be the best predictor to generating two floxed alleles in *cis*; although higher Cas9 mRNA concentration seemed to be another predictor, its effect was marginal.

**Table 2:** Generalized regression analysis to identify factors predicting the success of the 2-sgRNA 2-ssODN method

	Estimate	Standard error	*t* value	*p* value
Intercept	− 0.14	0.15	0.91	0.37
Efficiency of 5′ sgRNA	0.19	0.23	0.84	0.4
Efficiency of 3′ sgRNA	− 0.31	0.22	− 1.36	0.18
5′ *LoxP* insertion	0.01	0.92	0.017	0.98
3′ *LoxP* insertion	0.01	0.38	0.27	0.79
5′ *LoxP* × 3′ *LoxP* insertion	4.05	2.32	1.74	0.09
Cas9 mRNA concentration	0.004	0.002	1.92	0.06*
Cas9 protein concentration	0.0003	0.003	0.1	0.91
SgRNA concentration	− 0.002	0.002	− 1.12	0.27
ssODN concentration	0.002	0.001	0.13	21
Distance	− 0.00001	< 0.0001	− 1.28	0.21

***p < 0.01, *p < 0.05

To further determine if the factors such as nucleotide composition of the donor ssODN, ssODN length, or reagent concentration might explain the success of this approach, we applied a machine learning algorithm (random forests) on all loci that were correctly or incorrectly targeted [21]. Cross-validation of the machine learning model by bootstrapping aggregation (aka “out-of-bag error”), we did not find any association between the success of this approach and the concentration of the reagents (*p* value = 0.84), 5′ and 3′ ssODN donor length (respectively *p* values of 0.21 and 0.18) or the nucleotide compositions at the target site. Overall, we observed a low performance of the model when including ssODN donor length, nucleotide composition at the target site, and concentration of the reagents with an “out-of-bag estimate value” at 0.222. In summary, the assessment of regression combined with machine learning analysis could not clearly identify any specific factors contributing to the inefficiency of the two-donor floxing method. 

### Effect of microinjection skill factor on the two donor floxing method's outcome 

Because microinjection is one of the most critical steps in mouse genome editing experiments, we postulated that the efficiency of this method might depend on the skills of the technical personnel that perform microinjection. If skill influences the outcome (e.g., successful floxing alleles), we would be able to determine a difference between laboratories. We assessed this parameter by calculating the correlation between the laboratory as a predictive value and the positive outcome (successful floxed alleles). We did not find any evidence of a “skill” effect influencing the positive outcome (Kruskal-Wallis rank-sum test, chi-squared = 22, *p* = 0.16).

As an independent analysis for microinjection skill effect, we gathered another type of large dataset (creating knockin models using one-ssODN donor) from our consortium that gauged the microinjection skills of the technical personnel in our consortium. The 20 laboratories that participated in this study (including the ANU laboratory that only tested the *Mecp2* locus) had a nearly 90% success rate in generating one-ssODN donor knockin models; 293 out of 330 loci attempted were successful with an overall efficiency of 13% of live-born mice carrying the desired knockin alleles (Additional file 5: Table S5), suggesting that all of the laboratories that participated in the study had sufficient microinjection skills to create models using the CRISPR tool. Therefore, the lack of success in generating floxed alleles using the two-donor floxing method at the 20 participating laboratories was not due to the lack of technical skills in zygote-microinjection of CRISPR reagents.

### Assessing the efficiencies of other methods of generating conditional alleles

In recent years, a few other methods of generating conditional alleles have been described utilizing one-donor DNA, such as *Easi*-CRISPR (efficient additions with ssDNA inserts-CRISPR) or CLICK (CRISPR with lssDNA inducing cKO alleles) or double-stranded DNA (dsDNA)-based methods [17, 22, 23]. We hypothesized that one of the reasons for the lower efficiency of the two-donor floxing method could be that it requires two recombination events (each of which is independently subject to mutational events) whereas just one recombination event is sufficient for “one-donor DNA” methods. To test this postulate, we used the one-donor DNA method of generating a conditional allele in several loci that initially failed using the two-donor floxing method. Of the 61 independent targeting projects (for 56 loci), 48 projects failed with the two-donor method. Nine of these projects were then repeated using long single-stranded DNA donor approaches (4 with *Easi-*CRISPR method, 5 with CLICK method), 2 projects with dsDNA donor method, and 1 project with traditional ES cell targeting. Remarkably, all of the one-donor methods led to a successful generation of the floxed alleles (Additional file 6: Table S6). Of the 11 projects using Easi-CRISPR, CLICK, or dsDNA methods, we found the average success rate was 18.3% ± 13% with a median of 13.2%, which corresponds to an average 20-fold improvement over the two-donor floxing method (Kolmogorov-Smirnov test *p* value < 10^−5^). We examined whether this 18.3% average success rate could be due to a higher cleavage efficiency of the sgRNAs. Our analysis of the 5′ and 3′ guide cleavage efficiencies (by counting indel or *LoxP* insertion events) indicates that it is not the case (Mann-Whitney *U* test with a respective *p* value of 0.43 and 1 suggests that the difference in editing efficiency is not the reason for this discrepancy in success rate between these methods).

A possible explanation for the large difference in efficiency between the two-donor floxing method and those that use one-donor DNA is that the latter requires only one recombination event whereas the two-donor floxing method relies on two recombination events occurring in *cis*. If this hypothesis is true, we should observe a difference in the frequency of simultaneous insertion in 3′ and 5′ sites between these methods. To ascertain this hypothesis, we compared the frequency of simultaneous insertions of *LoxP* sites in 3′ and 5′ for Easi-CRISPR, CLICK, or dsDNA delivery and two-donor floxing method, and indeed found a difference (6 ± 20% two-donor floxing vs 76 ± 27% for other methods; Mann-Whitney *U* test *W* = 1, *p* value = 4.7 × 10^−5^) confirming our hypothesis.

Two modifications to the two-donor floxing method have been reported recently. The first modification involves the insertion of the second *LoxP* site via a second injection of reagents into the zygotes derived from the mouse lines of the first injection containing only one of the two *LoxP* insertions [24]. We refer to this method as “second *LoxP* insertion in the next generation.” The second modification involves introducing *LoxP* sites at two separate intervals in the same zygotes; the first one at the 1-cell stage and the second at the 2-cell stage [25]. We refer to this method as “sequential delivery of *LoxP* sites.” We tested 7 (of the 48 failed) projects using the “second *LoxP* insertion in the next generation” (Additional file 6: Table S6) method, and all resulted in the successful generation of floxed alleles. We found 14 ± 6% and 27 ± 32% efficiencies, respectively, in the first and the second *LoxP* insertion experiments, which indicate that the frequency of recombination is much higher when insertion of just one *LoxP* is treated as a separate event. In other words, the efficiencies of “one-donor” insertion events (individual events) are significantly higher than the combined efficiency of insertion of two-donors. Even though this approach takes nearly 1 year to complete, the method ultimately generates floxed alleles at an efficiency equivalent to “one-donor approaches” such as Easi-CRISPR, CLICK, or delivery of dsDNA. We also tested “sequential delivery approach,” the second modification of the two-donor floxing method introduced above, on three new loci, but none produced conditional alleles (Additional file 7: Table S7). We note that we only tested the microinjection mode of delivery to evaluate the sequential delivery approach. Considering that electroporation is regarded as less harsh approach (as it maintains a sufficiently reasonable amount of zygote viability after the two consecutive rounds of reagent introduction), we suggest that this method may require further evaluation at additional loci to draw a conclusion on its efficiency.

## Discussion

CRISPR-Cas9 technology has greatly facilitated the generation of mouse lines containing knockout or knockin alleles [26, 27]. However, the generation of conditional alleles remains a challenge using traditional ES cells and CRISPR-Cas9 gene-editing technologies. A previous report demonstrated 16% efficiency with two chimeric sgRNAs and two single-stranded oligonucleotides (referred here as two-donor floxing method) to produce conditional alleles in mice [11].

To evaluate the efficiency of the two-donor floxing method, we replicated the experiments described in the initial report on *Mecp2* (10) at three laboratories using the same experimental approaches to generate the sgRNA and Cas9 and microinjected into mouse zygotes along with ssODN donors. Although we observed single *LoxP* site insertions and *indels* at the cleavage sites, the method was unsuccessful in inserting two *LoxP* site in *cis*. These results prompted us to conduct a survey on the experiences of the global transgenic research community in using this method for the routine generation of cKO models. Twenty transgenic core facilities or large-scale knockout mouse centers participated in the consortium contributing data for 56 loci and over 17,000 microinjected or electroporated zygotes. In contrast to the 16% efficiency observed in the first report [11], the large dataset from the consortium suggests that the method is < 1% efficient and the method generally produces a series of undesired editing events, which occur at a nearly 100-fold higher rate than the correct insertion of the two *LoxP* sites in *cis*. These results are comparable with previous reports demonstrating an important disparity in success rate varying from 0 to 7% of mice harboring two *LoxP* sites insertions in *cis* whether delivered by microinjection [22, 25, 28–30] or by electroporation [25]. We and others also noted the large number of deletions at the target sites following DNA cleavage [22].

### What determines the success of the two-donor floxing method?

Because our dataset represented a “real-world situation” consisting of diverse experimental conditions, it actually provided an opportunity to investigate the effect of several different parameters on the method’s efficiency. The factors we analyzed included CRISPR-reagent formats; reagent concentrations, whether the guides were pre-tested or not; nucleotide composition at the target sites; nature of the loci (lethal or non-lethal); distance between the two guide cleavage sites; mouse strains used; microinjection technician skills; and laboratory/site factor. Our statistical and machine learning analyses suggested that none of these factors could explain the lower efficiency of this method. One plausible explanation is that the method relies on two recombination events leading to a successful insertion of two donors on the same chromosomal DNA, and the probability of such an event (among the multitude of other combination of events) becomes very low. We tested 11 loci (of the 48 failed projects with 2 ssODN donor approach) using one-donor DNA approaches (see the “What are the alternative approaches to the two-donor floxing method?” section for the list) with a 10- to 20-fold higher efficiency. This supports our hypothesis that the one recombination event that occurs when using one-donor DNA approach offers better efficiencies than two simultaneous recombination events needed when using the two-donor DNA approach*.* This raises a related question: will the efficiency be higher if *LoxP* insertions are far away from each other (e.g., several kilobases to 100 s of kilobases apart)? In this instance , because the two recombination events will be sufficiently far away from each other, will they negatively affect each other’s efficiency in a similar way as when they are close to each other? One study reported moderate efficiencies when they placed *LoxP* sites sufficiently far away, and the study included 6 loci [31]. The distances between the *LoxP* sites (and the efficiencies of insertions) were 361 kb (5%), 4 kb (2.5%), 205 kb (18%), 1.6 kb (5%), 348 kb (0%) ,and 7 kb (0%). We did not find evidence in our data to suggest that placing *LoxP* sites several kilobases apart will provide higher efficiencies, although our sample size is too small to formally rule out this hypothesis.

### What are the alternative approaches to the two-donor floxing method?

During the previous 2–3 years, a few strategies have been reported that offer potential alternatives to the low-efficiency two-donor floxing method. These newer methods use one-DNA donor formats including long single-stranded DNAs, linear dsDNAs, or circular dsDNAs (plasmids). The first set of alternative methods utilize long single-stranded DNA as a donor; a microinjection-based approach of this method was named *Easi*-CRISPR (efficient additions with ssDNA inserts-CRISPR) [23, 32], and an electroporation-based approach was named CLICK (CRISPR with lssDNA inducing cKO alleles) [17]. The efficiency of *Easi-*CRISPR for creating conditional alleles ranged from 8.5 to 18% with a median of 13% (in previous publications it ranged from 8.5 to 100% for seven different loci [23, 32]). The CLICK method was demonstrated using three loci (with four independent attempts) and had an efficiency ranging from 3.7 to 16.6% with a median of 11%. Along the lines of “one-donor DNA approaches,” the methods using two versions of double-stranded DNA donors have been reported, one each with linear and circular dsDNAs. A method termed Tild-CRISPR (targeted integration with linearized dsDNA-CRISPR) uses long-dsDNA as donors, which was demonstrated for two loci at 18.8% and 33.3% efficiency [33]. A second version of the dsDNA donor is a method utilizing circular dsDNA molecules (plasmids) to insert *LoxP* sites via pronuclear microinjection with efficiencies ranging from 1.5 to 5.9% for three loci [22], and 20% and 22% for 2 loci from our dataset.

We attempted seven of the loci that failed with the two-donor floxing method using the recently developed alternative methods such as the “second *LoxP* insertion in the next-generation method” where it uses the one-side *LoxP* inserted models of the first injection and re-injects the second side *LoxP* donor into zygotes derived from them. All of the projects produced successful conditional alleles at an average efficiency of 21%. The second method using the “sequential delivery of *LoxP* sites” introduces each of the guide RNA-ssODN sets into the same zygotes at 1-cell and 2-cell stages, respectively. We failed to generate cKO alleles for the three loci attempted, although the sample size was too small to provide any conclusion on the efficiency of this technique. Overall, our results suggest that the newer methods, particularly those that use the one-donor DNA approach, appear to be superior alternatives to the two-donor floxing method.

Based on these results, we make the following recommendations. Even though it is possible to obtain a cKO allele using the two-donor floxing method, because of its low efficiency, the method may not be suitable as the first choice for a routine generation of cKO mouse models. The newer methods, particularly those employing long DNA donors (ssDNA or dsDNA), provide superior efficiencies for the routine generation of cKO animal models.

### Reproducibility of CRISPR-based research methods

Genome editing tools utilizing the CRISPR-Cas systems have transformed many biomedical research fields as they have contributed to a number of powerful research methods. While many published methods are reproducible (as evidenced by their wide usage), the research community often encounters issues in reproducing some published methods. This may be because the original “proof-of-concept” papers have used underpowered studies in demonstrating the method, the results of which could be an exception, rather than the rule. Our community effort drawing upon the expertise and wealth of data from a multi-center transgenic mouse core facilities and research laboratories has allowed for the evaluation of collective experiences with the previously published methods of generating cKO mouse alleles. Our conclusions and recommendation of reproducible and efficient methods of genome editing will reduce wastage of resources, including animal lives. Our work exemplifies the importance of critical re-evaluation of the methods impacting larger research communities. Studies like this, where larger community experiences about the published methods are gathered and the large datasets are critically analyzed to make recommendations of best practices, can be vital, especially as the application of CRISPR-Cas9 technology continues to grow in both basic research and eventually into the clinic.

## Conclusion

In conclusion, we find the two-donor floxing method to be inherently biased for *indels* or substitutions at the double-strand break, deletion between the guide cleavage sites, or *trans*-insertion of the *LoxP* sites. Even though it is not impossible to obtain a cKO allele using the two-donor floxing method, because of its very low efficiency (~ 1200 zygotes were needed to generate 1 correctly targeted animal), the method may not be suitable for the routine generation of cKO mouse models. The method requires two simultaneous HDR events, an outcome that we find occurs very infrequently (< 1%) and is subject to unanticipated genome editing events at the cleavage sites. The newer methods, particularly those employing long DNA donors (ssDNA or dsDNA), offer higher efficiencies and, thus, are more suitable for the routine generation of cKO animal models.

## Material and methods

### Ethical statement

All experiments were approved by the respective Institutional Animal Care and Use Committees in the USA and Ethics Committees in Australia, Belgium, the Czech Republic, Japan, Spain, and the UK according to the guidelines or code of practice from the National Institute of Health in the USA, the National Health and Medical Research Council (NHMRC) in Australia, Animals (Scientific Procedures) Act 1986 in the UK, or Ministry of Education, Culture, Sports, Science and Technology (MEXT), The Ministry of Health, Labor and Welfare (MHLW) in Japan, the Central Commission for Animal Welfare (CCAW) in the Czech Republic, the Canadian Council on Animal Care (CCAC) in Canada, the National Ethics Code from the Royal Belgian (Flemish) Academy of Medicine in Belgium, and the European Code of Conduct for Research Integrity from All European Academies.

### *Mecp2* gene targeting using CRISPR-Cas9

*Mecp2* left single chimeric guide RNA (sgRNA) 5′-CCCAAGGATACAGTATCCTA-3′ and *Mecp2* right sgRNA 5′-AGGAGTGAGGTCTAGTACTT-3′ were designed as described in Yang et al. [11]. Ultramer oligonucleotides (Integrated DNA Technologies, Coralville, IA) were designed with sequences to T7 promoter for in vitro transcription, DNA target region, and chimeric RNA sequence. Complimentary oligos for each target sequence were annealed at 95 °C for 5 min, and the temperature was reduced 0.20 °C/s to 16 °C using a PCR machine (BioRad T100) before use as a template for sgRNA synthesis. sgRNAs were synthesized with the HiScribe™ T7 Quick High Yield RNA Synthesis Kit (New England Biolabs). Cas9 mRNA was obtained from Life Technologies or in vitro transcribed from a Chimeric pX330-U6-Chimeric-BB-CBh-hSpCas9 expression plasmid obtained from Addgene repository (Plasmid 42230; a donation from the laboratory of Feng Zhang). Given the lack of details on concentrations of CRISPR reagents used in Yang et al. floxing experiments [11], we chose 10 ng/μl sgRNA, 10 ng/μl Cas9 mRNA, and 10 ng/μl ssODN for the *Mecp2* floxing experiments based on the fact that these concentrations yielded high-efficiency ssODN insertions at all of the 12 loci attempted at the University of Nebraska transgenic core, the first of the three laboratories where *Mecp2* replication experiments were undertaken. Experiments with the same concentrations (10:10:10 ng/μl) were then repeated at two more laboratories (one in Australia and one in the Czech Republic).

### SgRNA design

SgRNAs were designed using available online tools such as CRISPOR, Chop-Chop, or CCTop [34, 35]. SgRNAs were cloned into pX330 and in vitro transcribed [27, 36, 37], or synthesized and annealed [38]. Cas9 mRNA or protein was purchased, in vitro transcribed, or purified in-house. Cas9 protein was complexed with the sgRNA or crRNA and the *trans*-activating crRNA [39] and then mixed with the ssODN prior to microinjection. Concentrations and site of injection for Cas9 protein or mRNA, sgRNA, and template repairs for each locus are indicated in Additional file 1: Table S1.

### Mouse husbandry, zygote microinjection and electroporation

Mice were purchased from various sources and maintained under specific pathogen-free conditions. Mice were maintained under 12/12-h light cycle, and food and water were provided ad libitum. Three to five week-old females were superovulated by intraperitoneal injection of pregnant mare’s serum gonadotropin (5 IU) followed by intraperitoneal injection of human chorionic gonadotropin hormone (5 IU) 48 h later. Superovulated females were mated with 8- to 20-week-old stud males. The mated females were euthanized the following day, and zygotes collected from their oviducts. Cytoplasmic or pronuclear injections were performed under an inverted microscope, using associated micromanipulators, and microinjection set-up. Electroporation of the embryos was performed with an electroporation device using a cuvette or 1-mm plate electrodes with the following parameters: 30-V square wave pulses with 100-ms interval using a BioRad electroporator device or four poring pulses (40 V, 3.5 ms, interval 50 ms, 10% voltage decay + polarity) followed by five to six transfer pulses (5 V, 50 ms, interval 50 ms, 40% voltage decay, alternating + and − polarity) using a NEPA21 electroporator device. Microinjected or electroporated zygotes were either surgically transferred into the ampulla of pseudo-pregnant females or cultured overnight at 37 °C and then surgically transferred at the 2-cell stage of development.

### Genotyping

As a general practice, at all centers, the genomic DNAs were first analyzed by PCR to identify mice containing both *LoxP* sites. The animals were declared negative if genotyping did not reveal the presence of shifted bands corresponding to the size of *LoxP* (34 bp). The DNA extraction was performed on ear punch or tail tip collected from mouse pups over 15 days old using a DNA extraction kit according to the manufacturer’s instructions. Primers were designed to amplify the regions encompassing the integrated *LoxP* sequence. PCR was performed using Taq polymerase under standard PCR conditions. The PCR products were then purified with ExoSAP-IT1 or a PCR Clean-Up System kit according to the manufacturer’s instructions. Sanger sequencing was performed in core facilities. To identify *LoxP* insertions, as a general practice at all centers, the two target sites were amplified individually to look for an increase in the amplicon size, which occurs if *LoxP* sites are inserted successfully. If the *LoxP* insertion was not observed in this first set of PCR analyses, the samples were declared negative for *LoxP* insertion, and in many such cases, the samples were not analyzed further (as the end goal of the project, i.e., generation of the floxed allele, was not met). In some cases, such samples were also sequenced to assess *indels* to understand if the guides were successful in cleaving the target site. In some cases, the entire regions encompassing both the guide cleavage sites were amplified to assess for deletions between the cleavage sites.

### Machine learning modeling

We used Python to prepare the dataset for modeling a random forest [21] classification model. This requires each target to be assigned a binary label, i.e., 0 or 1. We included each of the targets from Additional file 2: Table S2 for a total of 54 samples (49 unique loci). Due to the relatively low number of successes, we binned the data into two classes, “positives” and “negatives.” For this, we used the “correctly targeted” column from Additional file 2: Table S2. We assigned targets with 1 or more successful edits to positives and targets with zero successful edits to negatives. This resulted in 12 positives and 42 negatives.

We then generated a feature matrix for the dataset. This is a representation of the dataset in a format suitable for modeling using the random forest algorithm, where each row in the matrix represents a target. This involves converting properties from the target to a numerical representation. For example, we convert the DNA sequence AAATC to [A:0.6, T:0.2, C:0.2, G:0]. However, as well as single nucleotide proportions, we also include dinucleotide proportions (i.e., AA, AT, CT). We do this for each of the sequences (5′ guide RNA, 3′ guide RNA, 5′ donor sequence, and 3′ donor sequence) from Additional file 1: Table S1. We also include the lengths of the 5′ and 3′ donor sequence for each target.

With the labels and feature matrix, we then trained a random forest model using scikit-learn [40]. We trained the model using the default parameters. However, due to the imbalanced classes (a low number of 1 s vs. 0 s), we instructed the algorithm to use a custom “class_weights” parameter.

To quantify the model’s performance, we take advantage of the out-of-bag (OOB) error property of random forests. This value is generated during training by evaluating each “tree” in the random forest model using the samples that were not included for training that tree (through bootstrapping). We observe an OOB error of 0.222. Finally, we can identify important features using the “feature_importances_” property of the random forest model.

Source code is available on GitHub repository https://gist.github.com/aydun1/932f526867f7f8139b8e8eae7c76e866 and Zenodo doi: 10.5281/zenodo.3339039 under the MIT license.

### Statistics

To determine the statistical differences between the proportions, we performed a Fisher exact test or a Kruskal-Wallis sum-rank test or a Kolmogorov-Smirnov test. For means, we performed a Mann-Whitney *U* test. A generalized linear model calculation was performed with success of the two-donor floxing method as a response. Predictive variables were efficiency of the sgRNA; probability of *LoxP* insertions in 5′ and 3′ (5′_*LoxP* and 3′_*LoxP*); simultaneous insertion of the two *LoxP* sites (interaction between 5′_*Loxp* and 3′_*LoxP*); Cas9 mRNA, protein, plasmid, and ssODN concentrations; and distance between the distal and proximal target sites. Variance for each predictor was determined by calculating the diagonal of the variance-covariance matrix. Effect sizes and type II error were determined using Cohen effect size *d* statistics and power calculation. All statistical analyses were performed using Rstudio v1.1.423. The results were considered statistically significant at *p* < 0.05.

## Additional files

Additional file 1: Table S1. The guide RNA and singlestranded Oligonucleotide DNA sequences, their concentrations and the length of genomic regions floxed (in bp) reported in this study. (XLSX 29 kb)

Additional file 2: Table S2. The results of the two-donor floxing approach via microinjection for 49 unique loci. (XLSX 21 kb)

Additional file 3: Table S3. The results of the two-donor floxing approach via electroporation for 7 unique loci. (XLSX 11 kb)

Additional file 4: Table S4. Overall efficiency of the two-donor (2sgRNA-2ssODN) floxing method of generating the cKO alleles (XLSX 9 kb)

Additional file 5: Table S5. The details and the results of the 330 unique loci attempted for ssODN knock-in projects (i.e. point mutation knock-in or short-tag insertion projects). (XLSX 14 kb)

Additional file 6: Table S6. The details and the results of alternate methods of floxing, tested on 18 loci. (XLSX 23 kb)

Additional file 7: Table S7. The Evaluation of the modified method of two-donor floxing method (sequential delivery approach) reported in the Horii et al. 2017 report. (XLSX 11 kb)

## Data Availability

All data generated and/or analyzed during this study are included in this published article and its supplementary information files (Additional files 1, 2, 3, 4, 5, 6, and 7). Source code of the random forest model is available on GitHub repository https://gist.github.com/aydun1/932f526867f7f8139b8e8eae7c76e866 and Zenodo doi: 10.5281/zenodo.3339039 under the MIT license [41].
